# ACRE, a class of AP2/ERF transcription factors, activates the expression of sweet potato *ß-amylase* and *sporamin* genes through the sugar-responsible element CMSRE-1

**DOI:** 10.1007/s11103-024-01450-z

**Published:** 2024-05-07

**Authors:** Kenichiro Maeo, Yuki Nakaya, Nobutaka Mitsuda, Sumie Ishiguro

**Affiliations:** 1 Graduate School of Bioagricultural Sciences, Nagoya University, Furo-Cho, Chikusa-Ku, Nagoya, Aichi 464-8601 Japan; 2 Bioproduction Research Institute, National Institute of Advanced Industrial Science and Technology (AIST), Central 6, Higashi 1-1-1, Tsukuba, Ibaraki 305-8566 Japan

**Keywords:** Transcription factor, Gene expressions, Sugar induction, AP2/ERF, *Arabidopsis thaliana*, *Ipomoea nil*

## Abstract

Sugars, synthesized by photosynthesis in source organs, are loaded and utilized as an energy source and carbon skeleton in sink organs, and also known to be important signal molecules regulating gene expression in higher plants. The expression of genes coding for *sporamin* and *β-amylase*, the two most abundant proteins in storage roots of sweet potato, is coordinately induced by sugars. We previously reported on the identification of the carbohydrate metabolic signal-responsible element-1 (CMSRE-1) essential for the sugar-responsible expression of two genes. However, transcription factors that bind to this sequence have not been identified. In this study, we performed yeast one-hybrid screening using the sugar-responsible minimal promoter region of the ß-amylase gene as bait and a library composed only transcription factor cDNAs of *Arabidopsis*. Two clones, named Activator protein binding to CMSRE-1 (ACRE), encoding AP2/ERF transcription factors were isolated. ACRE showed transactivation activity of the sugar-responsible minimal promoter in a CMSRE-1-dependent manner in *Arabidopsis* protoplasts. Electric mobility shift assay (EMSA) using recombinant proteins and transient co-expression assay in *Arabidopsis* protoplasts revealed that ACRE could actually act to the CMSRE-1. Among the DEHYDRATION -RESPONSIVE ELEMENT BINDING FACTOR (DREB) subfamily, almost all homologs including ACRE, could act on the DRE, while only three ACREs could act to the CMSRE-1. Moreover, ACRE-homologs of Japanese morning glory also have the same property of DNA-binding preference and transactivation activity through the CMSRE-1. These findings suggested that ACRE plays an important role in the mechanism regulating the sugar-responsible gene expression through the CMSRE-1 conserved across plant species.

**Supplementary Information:**

The online version contains supplementary material available at 10.1007/s11103-024-01450-z.

## Key message

We identified transcription factors, ACREs, that bind to the CMSRE-1, essential for sugarresponsive gene expression, and activate in a CMSRE-1-dependent manner, and ACREs may be widely conserved among plant species.

**Supplementary Information:**

The online version contains supplementary material available at 10.1007/s11103-024-01450-z.

## Introduction

In higher plants, sugars are synthesized in photosynthetic source tissues such as mature leaves, translocated to sink tissues such as roots, fruits, seeds, and tubers generally in the form of sucrose, and utilized as a source of energy and carbon skeleton for their growth and development. Sugars are also translocated to newly formed reproductive organs, where they are converted into starch, proteins and lipids, and stored as various nutrients to support the growth of the next generation. Moreover, it is known that sugars act as signaling molecules that regulate many cellular processes, such as controlling gene expression and cell division, affecting the impacts of light and plant hormones, and determing the timing and frequency of organogenesis (Wind et al. 2010; Li et al. 2016; Sakr et al. 2019). It has been proven that the sugar-signaling pathway is extremely complex, with the existence of multiple pathways and cross talking with other signaling pathways such as plant hormones (Price et al. 2004; Sakr et al. 2019; Sami et al. 2019).

Although phenomena in response to sugars are observed in various species other than plants, few of the components involved in this signaling pathway have been reported. Moreover, with the exception of some kinases, such as Snf1-related protein kinase (SnRK)/AMP-activated protein kinase (AMPK) and TARGET OF RAPAMYCIN (TOR) kinase (Crepin and Rolland 2019; Jamsheer K et al. 2019), little is known about the components of this pathway conserved across species. For transcription factors (TFs) in plants in particular, there are no homologs to TFs known to mediate the sugar signaling in other organisms (Coccetti et al. 2018; Lagree et al. 2020), and it is considered that plant-specific TFs are involved in these regulatory mechanisms.

There are several plant-specific TF families, such as APETALA2/ETHYLENE RESPONSE FACTOR (AP2/ERF), VIVIPAROUS1/ABA INSENSITIVE3, AUXIN RESPONSE FACTOR, NAC and GRAS family, and some of these have plant-specific DNA-binding domains, such as AP2, B3, and NAC, which are conserved in each family (Riechmann and Ratcliffe 2000; Iida et al. 2005; Swarbreck et al. 2008; Pruneda-Paz et al. 2014). The AP2/ERF superfamily is defined by the AP2 domain, a DNA-binding domain consisting of 40–70 amino acids, and is roughly classified into three groups: the AP2 family containing two AP2 domains, the ERF family having a single AP2 domain, and the RAV family containing the B3 domain, a DNA-binding domain conserved in other plant specific TFs such as VP1/ABI3, in addition to single AP2 domain. The ERF family has further divided into the ERF subfamily (group B1 to B6) and the DEHYDRATION -RESPONSIVE ELEMENT BINDING FACTOR/C-REPEAT -BINDING FACTOR (DREB/CBF) subfamily (group A1 to A6), which were later redesignated as group I to X (Sakuma et al. 2002; Nakano et al. 2006). It has been reported that AP2/ERF TFs regulate the transcription of genes involved in numerous abiotic stresses such as salt, drought, heat, cold, and freezing (Akhtar et al. 2012; Xie et al. 2019).

The AP2/ERF TFs act on the *cis*-element in the target gene promoter through the AP2 DNA-binding domain to regulate target gene expression. The binding preference of AP2/ERF TFs has been revealed. DREB TFs involved in resistances to drought, cold and abiotic stresses (Shinozaki and Yamaguchi-Shinozaki 2000; Guo and Ecker 2004; Franco-Zorrilla et al. 2014) binds to Dehydration-Responsible Element (DRE), also known as C-repeat (CRT) with a A/GCCGAC core sequence, ERF TFs bind to Ethylene-Responsive Element (ERE) with an AGCCGCC core sequence, known as GCC-box; and several AP2/ERF TFs bind to both DRE/CRT and ERE (Franco-Zorrilla et al. 2014; Phukan et al. 2017; Xie et al. 2019). Meanwhile, ABA-INSENSITIVE4 (ABI4) binds to Coupling Element 1 (CE1) with a CACCG core sequence (Wind et al. 2012). Some amino acid residues in the AP2 domain involved in DNA binding have been indicated. For instance, Val14 and Glu19 of the AP2 domain, which differ between DREBs and ERFs, are important for determining the DNA-binding specificity of DREB TFs, whereas Ara37, conserved in the AP2/ERF family, is essential for binding to DRE and GCC-box (Sakuma et al. 2002; Liu et al. 2006). The 3D solution structure of the AP2 DNA-binding domain of AtERF1 with a GCC-box identified some amino acid residues that form contacts with DNA bases (Allen et al. 1998). However, these amino acids are highly conserved among members of the AP2/ERF family. Regarding group IXa of the ERF subfamily, it has been shown that amino acid residues in the first two ß-sheets of the AP2 DNA-binding domain contribute to the specificity and affinity of DNA-binding (Shoji et al. 2013; Chen et al. 2020; Qiao et al. 2022). Owing to the limited variety of binding sequences, the specificity of the DNA-binding sequence cannot explain the diversity of functions in regulating gene expression in response to various stimuli by AP2/ERF TFs.

Sporamin and β-amylase are the two most abundant proteins in the storage roots of sweet potato (*Ipomoea batatas*). Besides the developmentally regulated expression in storage roots, the expression of genes encoding *sporamin* and *β-amylase* is coordinately induced in vegetative tissues such as stem, leaf and petiole by high levels of metabolizable sugars, such as glucose and sucrose (Nakamura et al. 1991). We anticipated that the mechanism of regulating the expression of these two genes is one of the processes occurring under differentiation into storage organs, and is important for understanding the mechanism of regulating nutrient storage in plants.

To study the mechanism controlling the expression of *sporamin* and *ß-amylase* genes at the molecular level, we performed promoter analysis of these genes using transgenic tobacco plants. We identified sugar-responsible minimal promoters in both genes and named them *Spo*^*min*^ in the *sporamin* gene and *BAD430/435* in the *ß-amylase* gene. Moreover, we found that a *cis* element sequence, TGGACGG, designated carbohydrate metabolic signal-responsible element-1 (CMSRE-1), was conserved in both minimal promoters (Maeo et al. 2001; Morikami et al. 2005a). We attempted to identify TFs that act on this region by using several methods such as yeast-one-hybrid assay using these minimal promoters as bait, enhancer tagging using the reporter gene driven by the minimal promoters, and the screening of mutants exhibiting abnormal expression of sugar-responsible genes are abnormal (Morikami et al. 2005b). However, no TFs acting on CMSRE-1 sequence have been isolated.

In this study, we isolated several cDNA clones of *Arabidopsis* by yeast one-hybrid screening using the sugar-responsible minimal promoter region of the sweet potato *ß-amylase* gene as bait and a library composed only TF cDNAs of *Arabidopsis thaliana*. We have found two of the several positive clones with cDNAs encoding group II/A-5 of the DREB AP2/ERF subfamily TFs, designated the Activator protein binding to CMSRE-1 1 and 2 (ACRE1 and 2). These are highly homologous to each other and to ORA47 (referred to as “ACRE3” in this paper), which is reported to regulate jasmonic acid and abscisic acid biosynthesis and signaling (Chen et al. 2016). ACRE1 and ACRE2 have transactivation activity of *Spo*^*min*^ and *BAD* reporters in a CMSRE-1-dependent manner in *Arabidopsis* protoplasts. Electric mobility shift assay (EMSA) using recombinant MBP-fusion protein and transient co-expression assay in *Arabidopsis* protoplasts revealed that only ACRE1, 2 and 3 could act on the CMSRE-1 sequence as well as the DRE sequence, while other ACRE-like proteins of the group II/A-5 could act through the DRE sequence, but not to the CMSRE-1 sequence. Moreover, ACRE-homologs of Japanese morning glory (*Ipomoea nil*) show the same preference of binding sequence and transactivation activity. These results suggest that the CMSRE-1 and ACRE module is involved in the mechanism regulating sugar-responsible gene expression that functions across plant species.

## Materials and methods

### Yeast one-hybrid screening

The double and quadruple tandem repeats of the sugar-responsible minimal promoter region of the gene *ß-amylase* of sweet potato, BAD430 and BAD495 respectively (Maeo et al. 2001), were cloned into the pHISi vector (CLONTECH). Yeast strains in which the bait gene was integrated into the genome were prepared from strain YM4271 (CLONTECH) by homologous recombination between *HIS3* loci in the genome and the constructed plasmids. For Y1H, the library composed only of TFs in *Arabidopsis* was used (Mitsuda et al. 2010).

### Plant materials and expression analysis

*A. thaliana* (L.) Heynh. (ecotype Col-0) or the sGsL line of Col-0 harboring one copy of the *Spo*^*min*^*:GUS-Spo*^*min*^*:LUC* dual reporter (Tsukagoshi et al. 2005) was used. Growth, sugar treatment, and detection luminescence imaging of LUC activity were performed as previously described (Tsukagoshi et al. 2005). Expression analysis by RT-qPCR was performed as previously described (Kojima et al. 2024).

### Molecular cloning

The full-length cDNAs of ACREs, ACRELs, and homologs of morning glory were cloned into the pDONR201 vector using gateway technology (Invitrogen). For ACRE1 and ACRE2, plasmids purified from yeast growing on the selection media are used, while for the others, PCR products synthesized using attB sequence-attached primers (Suppl. Table S2), were applied. For transformation of the sGsL plants, the full-length cDNAs were placed downstream of the CaMV 35S promoter in the binary vector pGWB502Ω (Nakagawa et al. 2009) by Gateway cloning technology (Invitrogen). For effector plasmids of transient expression in protoplasts, a vector pUGW2 (Nakagawa et al. 2007) was used.

### Recombinant protein and electrophoretic mobility shift assay (EMSA)

For all TFs, full-length coding sequences were fused to the C-terminal part of MBP and purified from cells using amylose resin (New England BioLabs) in according with the manufacturer’s instructions with slight modifications. The recombinant MBP-TF fusion proteins were expressed in *E. coli* BL21-codonPlus (DE3)-RIL (Stratagene) using the modified *pDEST15* vector, in which the coding sequence of GST had been exchanged to those of MBP. *E. coli* cells expressing MBP-TF fusion protein were grown to the mid-log phase in Luria–Bertani broth containing ampicillin at 37 °C and then for 3 to 24 h at 30 °C in the presence of 1 to 5 mM isopropyl β-d-1-thiogalactopyranoside. Cells collected by centrifugation were suspended in Column Buffer containing 0.5 mM PMSF. Cells were disrupted by a sonicator (Ohtake works). After centrifugation, the lysate was mixed with one-tenth volume of amylose resin to bind MBP-TF. The binding reaction was performed by rotation at 4 °C overnight, and then MBP-TF fusion protein was eluted with Column Buffer containing 10 mM maltose three times.

The EMSA was performed as described previously (Maeo et al. 2009) with slight modifications. The standard binding reaction (20 µL) contained 15 mM HEPES–KOH (pH7.5), 25 mM KCl, 0.25 mM EDTA, 6% glycerol, 2 mM dithiothreitol, and 20 fg of DNA probe labeled with BIO-ON (Sigma) by PCR reaction using end-labeled primers and recombinant protein (0.2 to 0.8 µg). The reaction mixture was incubated at room temperature for 30 min, and analyzed by polyacrylamide gel electrophoresis followed by detection with a chemiluminescence using chemiluminescent nucleic acid detection module kit (Thermo Fisher Scientific).

### Systematic evolution of ligands by exponential enrichment (SELEX)

Systematic Evolution of Ligands by Exponential Enrichment (SELEX) was performed as described previously (Haga et al. 2011; Santuari et al. 2016). MBP-TF fusion proteins binding to amylose resin were prepared as described above. Resins were washed extensively with Column Buffer. An oligonucleotide mixture containing a random sequence of 26 nucleotides flanked by 24-nucleotide primer sequences on both sides (5′-AGCATCACTGATTCAAGAGCATAG-N26-TTCACCTTCAGAACTGATGTACTC-3′) was converted into double-stranded DNA by PCR with PrimeSTAR HS DNA polymerase (Takara Bio) and used as R74 oligonucleotides. Amylose resin bound with MBP-TFs was mixed with 100 ng of R74 oligonucleotides in binding buffer composed of 15 mM HEPES–KOH (pH 7.5), 6% glycerol, 2 mM DTT, 75 mM KCl, 0.5 mM EDTA, 50 ng/mL poly(dI-dC), and 0.5 mM PMSF at room temperature for 30 min with gentle shaking. Resins were washed with the Column Buffer, and DNA was recovered from the beads by extraction with phenol:chloroform and ethanol precipitation. After amplification by PCR, 10 ng of DNA was subjected to the next round of selection by binding with MBP-TF amylose resin. After the selection step had been repeated 10 times, DNA fragments recovered from the resin were cloned into the *Eco*RV site of pBlueScript I KS( +) (Stratagene) using *E. coli* DH5α. Direct PCR for more than 50 insertion-positive colonies was performed and their DNA sequences were determined.

### Transient expression assay in *Arabidopsis* protoplasts

Preparation of protoplasts from the *Arabidopsis* T87 suspension cultured cells and transient transformation of protoplasts by a modified version of the polyethylene glycol method were performed as described previously (Maeo et al. 2009). The oligonucleotides containing each element were cloned into the *Xba*I site of the *35S46:LUC* vector (Inaba et al. 2000), making various reporter plasmids. An empty vector without a coding sequence of TFs, *p35Snos*, and *35S:hRLUC* plasmid for the expression of hRLUC were used as a negative control and an internal control, respectively.

A suspension of protoplasts (150 µL; 10^6^ protoplasts per mL) was co-transfected with 15 µg each of the *LUC* reporter and the effector plasmid. The protoplasts were incubated at 22 °C for 20 h before collection and measurement of reporter activities. The LUC and hRLUC activities were measured using the Dual-Luciferase Reporter Assay system (Promega) and Luminoskan Ascent (Thermo Scientific). The LUC activity was normalized according to the hRLUC activity in each assay, and the relative ratio was determined.

## Results

### Cloning of ACRE genes

**Fig. 1:**
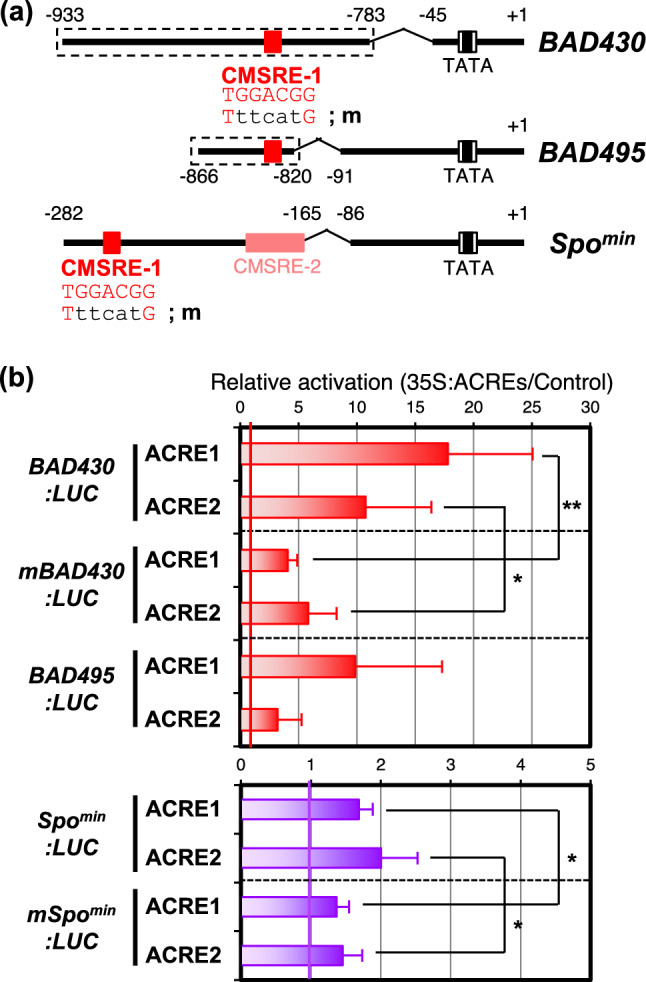
Transactivation of sugar-responsible minimal promoters by co-expression of ACRE1 and ACRE2. **a** A schematic structure of the sugar-responsible promoter of *ß-amylase* and *sporamin*, *BAD430:LUC*, *BAD495:LUC* and *Spo*^*min*^*:LUC*. Regions surrounded by a dotted line were used as bait in yeast one-hybrid screening. The CMSRE-2 is another element required for sugar-responsible expression of the *sporamin* gene (Morikami et al. 2005a). **b** Transactivation of sugar-responsible minimal promoters by the co-expression of ACRE1 and ACRE2 in *Arabidopsis* protoplasts. The LUC activity in each assay was normalized to the hRLUC activity, and specific activity (LUC/hRLUC) is expressed relative to values obtained with the empty vector. Results represent the means of 6 to 10 independent experiments, with the error bar representing SD. Asterisks indicate significant change at *P < 0.05 and **P < 0.01 (Student’s t test) compared with the results with the wild-type reporters

To isolate the TFs that bind to the CMSRE-1 sequence that is essential for the sugar-responsible expression of *sporamin* and *ß-amylase* genes in sweet potato, we performed yeast one-hybrid screening. As bait, we chose two fragments, BA1 (from − 933 to − 783) and BA2 (from − 866 to − 820), which are portions of previously identified sugar-responsible minimal promoters (*BAD430* and *BAD495*) of *ß-amylase* gene (Fig. 1, Maeo et al. 2001). To increase the binding efficiency of target proteins, the BA1 and BA2 fragments in the bait plasmid were concatenated twice and four times, respectively. For the library, a TF-specific cDNA library consisting of about 1500 clones of known *Arabidopsis* TFs was used (Mitsuda et al. 2010). We identified 34 and 22 positive clones by the screening with 2xBA1:HISi bait and 4xBA2:HISi bait, respectively, and confirmed that all clones reproducibly grew well on the selective medium (Suppl. Table S1). The positive clones included three plant-specific B3 domain proteins such as FUS3 and LEC2 and five homeobox-containing ATHB proteins, both of which have been shown to act on the sugar-responsible minimal promoters through binding to the different *cis*-element from the CMSRE-1 (Morikami et al. 2005b). Among the rest, we identified two clones, corresponding to At5g21960 and At1g19210, which we designated as Activator protein binding to CMSRE-1 1 and 2 (ACRE1 and ACRE2), respectively, after we confirmed that they bound to CMSRE-1 (described below). Both genes belonged to the group II ERF, previously referred to as the group A-5 of the DREB subfamily, of the AP2/ERF-domain TF family (Nakano et al. 2006; Suppl. Fig. S1). Homology analysis of predicted amino acid sequences revealed that the 15 members of the group II ERF in *Arabidopsis* were separated into six DEARs (DEAR clade) and nine the others. In the latter ACRE1 and ACRE2 formed a single small clade (ACRE clade) with another closely related homolog, At1g74930, which has been known as octadecanoid-responsive AP2/ERF-domain transcription factor 47 (ORA47; Chen et al. 2016, hereafter called ACRE3 in this paper). We named other genes in the group II ERF ACRE-LIKE1 (ACREL1) to ACREL6 (Supp. Fig. S1). The DEAR proteins possessing an EAR motif in their C-terminus were shown to be transcriptional repressors (Tsutsui et al. 2009), whereas ORA47/ACRE3 was demonstrated to be an activator (Pauwels et al. 2008), suggesting that ACRE1 and ACRE2 might be activators. Although ORA47/ACRE3 has been shown to be involved in regulation of the genes for jasmonic acid and abscisic acid biosynthesis and signaling, physiological roles of ACRE1 and ACRE2 have not yet been reported. As for the function of ACRELs, ACREL4/ERF019 has been shown to be involved in plant growth and development, causing increased tolerance to drought (Scarpeci et al. 2017), and ACREL5/ERF014 has been shown to act as a regulator that modulates immunity (Zhang et al. 2016).

### ACRE1 and ACRE2 activate the expression of sugar-responsible minimal promoters containing CMSRE-1

To investigate whether ACRE1 and ACRE2 could regulate transcription from the sugar-responsible minimal promoters, we performed transient co-expression assays with LUC reporters driven by the sugar-responsible minimal promoters and forcibly expressed ACRE effectors in protoplasts derived from cultured *Arabidopsis* T87 cells (Maeo et al. 2009). ACRE1 and ACRE2 activated the expression of *BAD430:LUC* reporter more than 10 times higher than the level of no-effector control (Fig. 1b). The activation levels were significantly decreased when base substitutions were introduced into the CMSRE-1 sequence of *BAD430:LUC* reporter (*mBAD430:LUC*), suggesting that ACRE1 and ACRE2 activate the transcription from this promoter in a CMSRE-1-dependent manner (Fig. 1b). Similar activation was observed when *BAD495:LUC* was used as a reporter. Furthermore, ACRE1 and ACRE2 activated the expression from another sugar-responsible *Spo*^*min*^ promoter, although the transactivation levels were lower than the cases of *ß-amylase* promoters. Nevertheless, the expression levels were decreased when CMSRE-1 sequence in the promoter was disrupted, suggesting that the activation was CMSRE-1-dependent (Fig. 1b).

### ACREs activate the gene expression through CMSRE-1

**Fig. 2:**
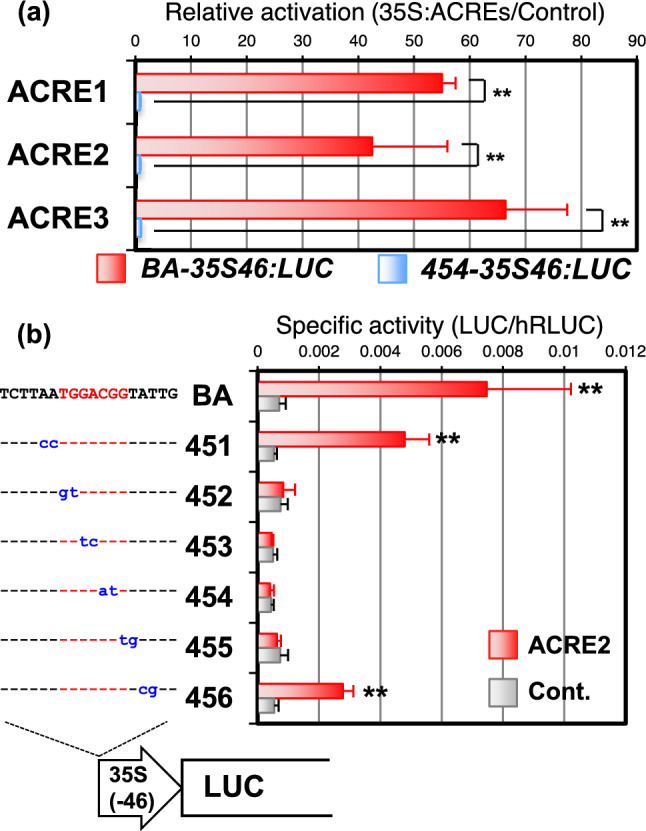
The CMSRE-1 is necessary for transactivation by ACRE2. Transactivation of *BA-35S46:LUC* with the serial base-substitution around the CMSRE-1 by co-expression of ACRE2 in Arabidopsis protoplasts. The average of relative activity (LUC/hRLUC) in 5 to 10 independent experiments is shown with the error bar representing SD. Asterisks indicate significant change at *P < 0.05 and **P < 0.01 (Student’s t test) with the control

The minimal promoters contained many sequence elements that might interact with ACREs for transactivation. To address whether the CMSRE-1 sequence is sufficient for the binding and transactivating activity of ACRE proteins, we prepared a reporter gene, in which an 18-bp DNA fragment comprising CMSRE-1 and its flanking sequence in sweet potato *ß-amylase* promoter (designated CMSRE-1-BA) was placed in front of the TATA-box element derived from cauliflower mosaic virus (CaMV) 35S promoter and the following LUC coding sequence (*BA-35S46:LUC*). All ACREs considerably activated the expression of the reporter gene, when we introduced the reporter plasmid and each ACRE effector plasmid into the *Arabidopsis* protoplasts (Fig. 2a). The transactivation was abolished when CMSRE-1 (TGGACGG) was changed to TGGAATG (*454-35S46:LUC*, see Fig. 2b), suggesting that the transcriptional activation by ACREs is CMSRE-1-dependent (Fig. 2a). To examine further the sequence specificity of ACRE transactivation, we introduced a series of 2-bp substitutions into the CMSRE-1 sequence (451–456, Fig. 2b) and employed ACRE2 as a representative of three ACREs. The ACRE2 never activated the reporters having substitutions in the CMSRE-1 sequence (452–455), while the LUC activity remained when only its flanking sequence was substituted (451 and 456). These results clearly showed that ACRE activates the transcription through CMSRE-1 and that the sequence is sufficient for the transactivation by ACRE. The sequence specificity was consistent with the results of promoter analysis for sugar-responsible expression in transgenic tobacco plants (Maeo et al. 2001).

### ACRE proteins bind to CMSRE-1 and DRE

**Fig. 3:**
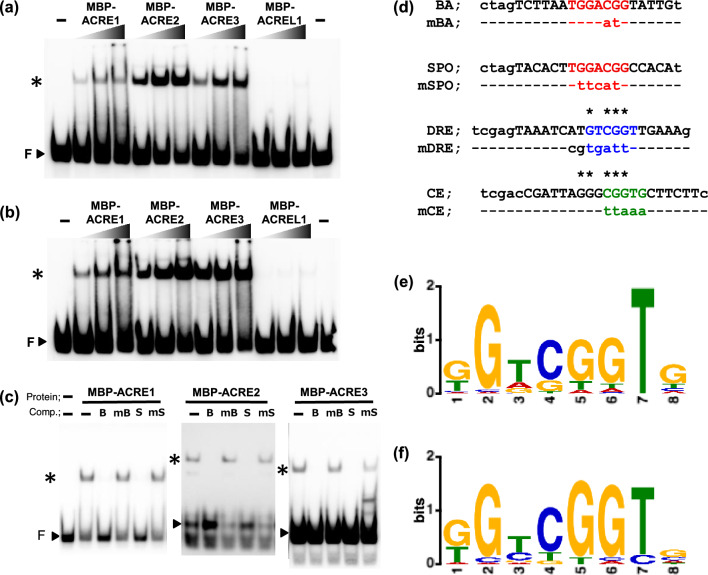
DNA-binding activity of the ACRE proteins. **a, b** The DNA-binding activity of ACRE and ACREL1 fused with MBP was analyzed by electrophoretic mobility shift assays using BA oligonucleotide (**a**) and SPO oligonucleotide (**b**) as probes. The amount of protein used in the assay were 0.2, 0.4, and 0.8 µg for each protein. **c** The specificity of binding activity of the ACRE2 protein. The MBP-ACRE1 (left panel), MBP-ACRE2 (middle panel), and MBP-ACRE3 (right panel) fusion proteins were incubated with 20 fmol of biotin-labeled BA probe in the absence ( −) or presence of a 100-fold molar excess of competitor oligonucleotides, BA (B), mBA (mB), SPO (S), mSPO (mS), DRE (D), and mDRE (mD). Arrowhead indicated by F shows the position of free probe. **d** The sequences of oligonucleotides used for EMSA. **e, f** The consensus binding sequence of ACRE1 (**e**) and ACRE3 (**f**) determined by SELEX. Consensus ACRE1- and ACRE3-binding sequences deduced from the nucleotide sequences of 44 and 48 oligonucleotides, respectively, selected by SELEX

To address the DNA-binding activity of ACRE proteins to CMSRE-1, recombinant ACRE1, ACRE2, ORA47/ACRE3 and ACREL1 fused with maltose binding protein (MBP) were expressed in *Escherichia coli*, purified by amylose-resin (Suppl. Fig. S2), and examined by EMSA. All MBP-ACRE fusion proteins retarded the electrophoretic mobilities of biotin-labeled oligonucleotide probes, BA and SPO, containing the CMSRE-1 and its flanking sequences in the sweet potato *ß-amylase* and *sporamin* minimal promoters, respectively (Fig. 3a, b and d). No shifted band was detected when MBP-ACREL1 fusion protein and MBP alone were tested (Fig. 3a, b, Suppl. Fig. S3). The shifted bands with MBP-ACRE and BA probe disappeared when we added excess amounts of unlabeled BA or SPO oligonucleotides as competitors, whereas mBA and mSPO competitors, in which CMSRE-1 was disrupted, did not affect the complex formation (Fig. 3c, d). These results showed that ACRE1, ACRE2 and ORA47/ACRE3 recognize and bind to the CMSRE-1 sequence. The competition experiments also revealed that the MBP-ACRE2 binds to the DRE sequence (Suppl. Fig. S3b), although only four nucleotides were in common between CMSRE-1 and DRE. The DRE-binding activity of all three ACREs was confirmed when DRE oligonucleotide was used as a probe (Suppl. Fig. S3b). Nevertheless, they did not bind to AtBA probe, which contained CMSRE-1-like sequence with a single base substitution derived from the promoter of *Arabidopsis β-amylase*, a sugar-responsive gene (Suppl. Fig. S3b, Mita et al. 1995), suggesting they have a strict sequence preference.

To clarify the binding preference of ACREs at the nucleotide level, we performed the systematic evolution of ligands by exponential enrichment (SELEX) assay (Tuerk and Gold 1990; Haga et al. 2011; Santuari et al. 2016). The consensus sequences for preferable binding of ACRE1 and ORA47/ACRE3 closely resembled and matched the nucleotides in common between CMSRE-1 and DRE (Fig. 3e, f).

The nucleotide sequence in the CMSRE-1 sequence required for activation of *BA-35S46:LUC* by ACRE2 was consistent with that required for sugar-induced expression of the minimal sugar-responsible promoter, *BADs* and *Spo*^*min*^, in the leaves of the transgenic tobacco plants (Fig. 1; Maeo et al. 2001; Morikami et al. 2005a). Additionally, this suggested that the CMSRE-1 sequence and ACRE acting on this sequence play important roles in regulating the sugar-responsible expression of genes encoding sporamin and ß-amylase, although there is as yet no direct evidence of this. To test for this possibility, we examined the expression of the *ACRE* gene in response to sugar by RT-qPCR; the expression of *ACRE1*, *ACRE2*, and *ACRE3* was not affected by sugar supply in *Arabidopsis* leaves (Suppl. Fig. S6).

### ACRELs do not activate the transcription through CMSRE-1 but DRE

**Fig. 4:**
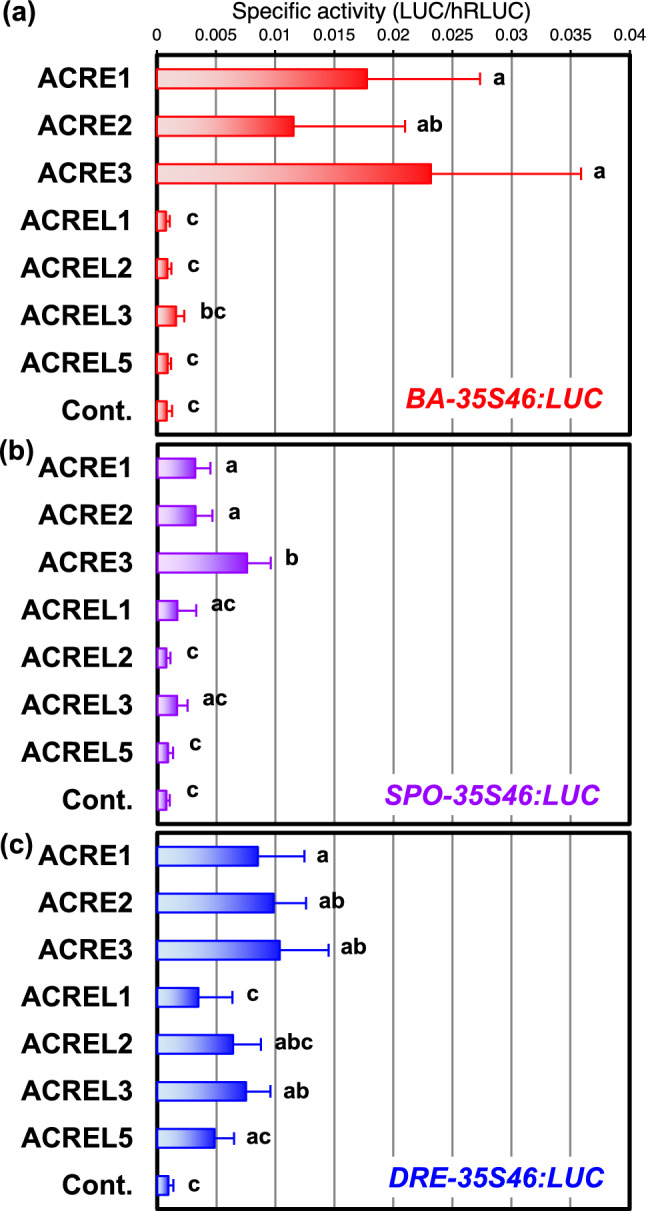
Transactivation of *BA-35S46:LUC* (**a**), *SPO-35S46:LUC* (**b**) and *DRE-35S46:LUC* (**c**) by co-expression of ACRE and ACRE-like in *Arabidopsis* protoplasts. The average relative activity (LUC/hRLUC) in 6 to 16 independent experiments is shown with the error bar representing SD. Values that were significantly different from one another according to Tukey’s multiple comparison test are indicated by different letters (P < 0.05)

As we previously described, the *Arabidopsis* genome contains six ACREL proteins forming a single clade with ACREs, suggesting that they might bind to CMSRE-1 and activate the transcription from the sequence. We carried out transactivation experiments with the *BA-35S46:LUC* reporter gene and ACRE and ACREL effector. ACRE1, 2, and 3 comparably activated the expression of the reporter gene, whereas ACREL3 only slightly activated and other examined ACREL genes, ACREL1, 2, and 5, never activated the transcription (Fig. 4a). Similar results were obtained when *SPO-35S46:LUC*, in which CMSRE-1-BA was replaced with another 18-bp sequence, CMSRE-1-SPO, consisting of CMSRE-1 and its flanking sequence in *sporamin* promoter, was used as a reporter (Fig. 4b), although the expression levels were lower than the results with CMSRE-1-BA. This suggested that the difference between the flanking sequences might affect the activation levels. In contrast, both ACREs and ACRELs comparably activated the expression when *DRE-35S46:LUC* was used as a reporter, in which DRE and its flanking sequence of *AtDGAT1* gene promoter were joined with the TATA -box element and LUC coding sequence (Fig. 4c). These results suggest that the examined ACREL proteins worked as transcriptional activators recognizing a DRE-containing promoter, but hardly ever or never activated the expression from CMSRE-1 containing promoters. In contrast, ACRE1, ACRE2, and ORA47/ACRE3 activated the transcription through both CMSRE-1 and DRE sequences. These sequence preferences agree well with the DNA-binding specificity of ACREL1 examined by EMSA (Fig. 3).

### Amino acid residue required for binding sequence specificity in AP2 domain

The DREB subfamily TFs contain a highly conserved DNA-binding AP2 domain in their sequences. It has been reported that a glutamic acid residue at the 19th position of the AP2 domain in *Arabidopsis* DREB1A and DREB2A is important to determine their sequence specificity of DNA binding (Cao et al. 2001; Sakuma et al. 2002). Since this residue is substituted to Leu in only three ACRE proteins, we hypothesized that the Leu residue provides the ability to bind to the CMSRE-1 sequence. To test this hypothesis, we substituted the corresponding Leu (Leu29) and Glu (Glu69) residues in ACRE2 and DREB1A to Glu and Leu residues, respectively (Suppl. Fig. S4a), and examined their transactivation of *BA-35S46:LUC* and *DRE-35S46:LUC* reporter by transient co-expression in *Arabidopsis* protoplasts. ACRE2 (L29E) showed a significantly decreased expression of *BA-35S46:LUC* in comparison to the case when unsubstituted ACRE2 was used as an effector, while both the substituted and unsubstituted ACRE2s provided similar transcription activities for *DRE-35S46:LUC*. This suggested that the substitution specifically affected the interaction with the CMSRE-1 sequence (Suppl. Fig. S4b). In contrast, DREB1A specifically activated the expression of *DRE-35S46:LUC* but never that of the *BA-35S46:LUC* reporter, indicating that its E68L substitution did not affect the specificity (Suppl. Fig. S4b). These results indicate that the 19th leucine residue in the AP2 domain of ACRE2 plays an important role in the specificity of the DNA-binding sequence, but is not decisive in determining the binding sequence specificity.

### DNA-binding activity of ACRE-homologs in Japanese morning glory

**Fig. 5:**
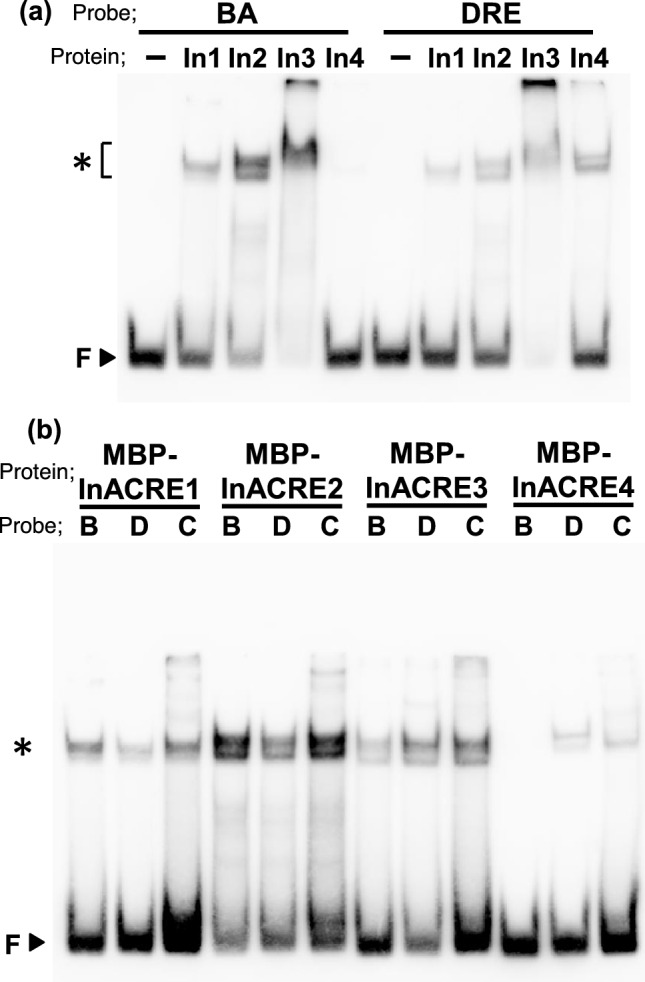
DNA-binding activity of the InACRE proteins. **a** The DNA-binding activity of InACREs fused with MBP was analyzed by electrophoretic mobility shift assays using BA and DRE oligonucleotide as probes. **b** The specificity of binding sequence of the InACRE proteins. The MBP-InACRE fusion proteins were incubated with 20 fmol of biotin-labeled BA (B), DRE (D) and CE (C) oligonucleotides shown in Fig. 2d, as probes. Arrowhead indicated by F shows the position of free probe

The CMSRE-1 sequence has been shown to be essential for sugar-inducible expression from *Spo*^*min*^ and *BAD* promoters in tobacco and *Arabidopsis* (Maeo et al. 2001; Morikami et al. 2005a, b). In sweet potato, it was reported that the *Spo*^*min*^ promoter-GUS gene was upregulated by exogenously supplied sucrose in leaves and stems and expressed highly and constitutively in storage roots (Honma and Yamakawa 2019). To investigate whether ACREs are conserved in sweet potato or its close relatives, we chose Japanese morning glory (*Ipomoea nil*), which is in the same genus as sweet potato, and identified ACRE orthologs in a morning glory genome database, AsagaoDB (http://ipomoeanil.nibb.ac.jp/) (Hoshino et al. 2016). By tBLASTn search against the database using the predicted amino acid sequence of ACRE1, we found six ESTs corresponding to INIL03g18104, INIL01g20129, INIL14g06668, INIL01g36561, INIL03g1810 and INIL02g10184 (Suppl. Fig. S1). Apart from two genes (INIL14g06668 and INIL01g3656, of which no cDNA clones were available), we obtained cDNA clones of the remaining four genes, INIL03g18104, INIL01g20129, INIL03g1810 and INIL02g10184, termed InACRE1 to 4, and analyzed DNA-binding and transactivation activities of the gene products. Recombinant InACRE1, 2, 3, and 4 fused with MBP (MBP-InACRE) were expressed in *E. coli* and fusion proteins were purified using amylose-resin (Suppl. Fig. S5). The binding activity of MBP-ACREs to the CMSRE-1 sequence was examined by EMSA. MBP-InACRE1, 2, and 3 fusion proteins formed shifted bands with both BA and DRE probes, whereas MBP-InACRE4 fusion protein did so only with the DRE probe (Fig. 5a). It has been shown that ABI4, belonging to the A3 group of the DREB subfamily, binds to the CE1 in promoter regions of its target genes in *Arabidopsis* (Wind et al. 2012). We examined whether InACREs could bind to the CE1 sequence. As with the DRE probe, all InACREs bound to the CE probe containing the CE1 sequence derived from the *ABI4* gene promoter (Fig. 5b).

### Transactivation of morning glory ACREs

**Fig. 6:**
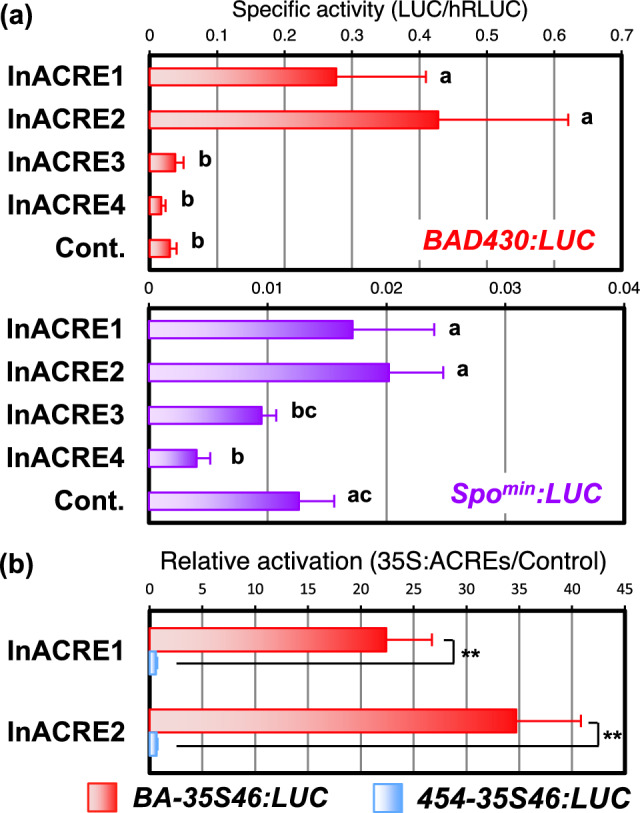
Transactivation of the sugar-responsible minimal promoters by the co-expression of InACRE in *Arabidopsis* protoplasts. **a** Transactivation of *BAD430:LUC* (upper panel) and *Spo*^*min*^*:LUC* (lower panel) by co-expression of InACRE in *Arabidopsis* protoplasts. The average of relative activity (LUC/hRLUC) in 5 to 6 independent experiments is shown with the error bar representing SD. Values that were significantly different from one another according to Tukey’s multiple comparison test are indicated by different letters (P < 0.05)

**Fig. 7:**
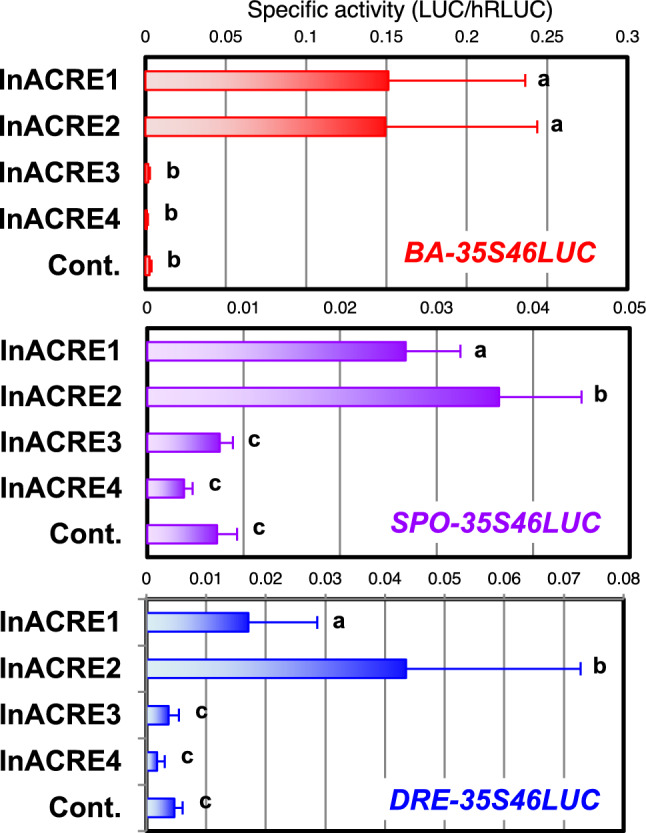
Transactivation through elements known to bind with group II ERF by co-expression of InACRE. Transactivation of *BA-35S46:LUC*, *SPO-35S46:LUC*, *DRE-35S46:LUC* and *CE-35S46:LUC* by co-expression of InACRE in *Arabidopsis* protoplasts. The average of specific activity (LUC/hRLUC) in 6 to 12 independent experiments is shown with the error bar representing SD. Values that were significantly different from one another according to Tukey’s multiple comparison test are indicated by different letters (P < 0.05)

Next, we investigated whether InACREs could regulate the transcription of the sugar-responsible minimal promoters. When *BAD430:LUC* was used as a reporter, InACRE1 and InACRE2 significantly increased the expression of the reporter genes whereas InACRE3 and InACRE4 did not show such enhancement (Fig. 6). To confirm the ability of InACREs to affect CMSRE-1, we used *BA-35S46:LUC*, *SPO-35S46:LUC*, and *DRE-35S46:LUC* as reporters for transactivation in protoplasts. InACRE1 and InACRE2 increased the expression of all reporters, but none was activated by InACRE3 or InACRE4 (Fig. 7). Furthermore, InACRE1 and InACRE2 did not activate the expression of the *454-35S46:LUC* reporter, indicating that this activation was CMSRE-1-dependent. Taken these findings together, we concluded that InACRE1 and InACRE2 activate transcription through both CMSRE-1 and DRE sequences, similar to ACRE1, ACRE2, and ORA47/ACRE3 of *Arabidopsis*.

### Sugar-responsible activation of *Arabidopsis* and sweet potato genes by ACREs

In order to examine the physiological function of ACREs, we prepared ACRE-overexpressing *Arabidopsis* plants. We found that the expression of *Atß-amylase* (*Atß-Amy*) gene (At4g15210), which has been identified as a sugar-responsible gene, was clearly upregulated in the plants that overexpressed any of *ACRE* genes (Suppl. Fig. S6a). In contrast, overexpression of *ACREL1* had no effect. The ACRE-dependent expression of *Atß-Amy* was enhanced when the plants were treated with 5% sucrose, indicating that ACREs mediated a responsivity to sugars (Suppl. Fig. S6a). We also tested the transactivation of *Atß-Amy:LUC* fusion gene in *Arabidopsis* protoplasts. All ACREs but not ACREL1 significantly activate the expression of the reporter (Suppl. Fig. S6b). Although we could not find obvious CMSRE-1 sequence in the promoter, the result indicated that ACREs activate the expression of *Atß-Amy* gene. Hence, we concluded that ACREs are involved in the sugar-responsible expression of endogenous genes in *Arabidopsis*.

To examine the involvement of ACREs in the sugar-responsible activation of *sporamin* promoter, we used sGsL line of *Arabidopsis* (Tsukagoshi et al. 2005). Overexpression of ACREs enhanced the LUC activity in sugar-dependent manner, confirming that ACREs mediate the sugar signal to the promoter (Suppl. Fig. S7). It should be noted that overexpression of ACREL1 also enhanced the expression of LUC reporter. An excess amount of ACREL1 may contribute to the activation of *sporamin* promoter, even though its binding affinity to CMSRE-1 was low.

## Disscussion

In this study, we identified two *Arabidopsis* TFs, ACRE1 and ACRE2, that interact with the previously reported sugar-responsible minimal promoters of the genes encoding ß-amylase and sporamin, both of which are two major storage proteins in sweet potato. In transient co-expression analysis in *Arabidopsis* protoplasts, ACRE1 and ACRE2 activated the transcription from *ß-amylase* and *sporamin* promoters. The transactivation was dependent on the CMSRE-1 sequence, which had been identified as the most important *cis*-element in both of the promoters (Maeo et al. 2001; Morikami et al. 2005a). A database analysis revealed that both proteins belong to the DREB A-5 (ERF group II) subfamily of AP2/ERF TFs and are close homologs of ORA47/ACRE3, which is proposed to possess jasmonic acid- and abscisic acid-related functions (Chen et al. 2016). Further characterization of ACRE TFs including ORA47/ACRE3 revealed that they recognize and bind to both CMSRE-1 (TGGACGG) and DRE (GTCGGC/T in the opposite strand), which share “GnCGG” core sequence. This was consistent with the results of SELEX experiments for ACRE1 and ACRE3. Moreover, we showed that they facilitate gene expression by activating the promoters containing either CMSRE-1 or DRE sequences. In contrast, ACREL proteins, as well as DREB1A, could activate the expression from only DRE-containing promoters. Hence, we concluded that ACRE1, ACRE2, and ORA47/ACRE3 are the TFs that activate the transcription from CMSRE-1 in *Arabidopsis*.

In EMSA using recombinant MBP-ACRE fusion proteins, we showed that ACRE1, ACRE2, and ORA47/ACRE3 bind to not only the CMSRE-1 sequence, a *cis*-element necessary for the sugar-responsible expression of *ß-amylase* and *sporamin* genes, but also the DRE sequence to which various members of the DREB subfamilies have been shown to bind. This was consistent with the results of SELEX assay, where the identified sequence was GnCGGT, which is a consensus sequence of CMSRE-1 and DRE. Note that the last T in the sequence is present at the next position of CMSRE-1 in the *ß-amylase* promoter but not in the *sporamin* promoter. This seems consistent with the relatively weak activation of SPO reporters in the transactivation by ACRE proteins. Nevertheless, the competition assay in EMSA revealed that ACRE bound to BA and SPO sequences in a similar fashion. The binding sequence specificities of ERF017/ACRE2 and ERF018/ORA47/ACRE3 were determined previously in high-throughput analyses using protein-binding microarrays and DNA affinity purification sequencing (Franco-Zorrilla et al. 2014; O’Malley et al. 2016). According to the database that summarized the results (http://jaspar.genereg.net/), ERF017/ACRE2 and ERF018/ORA47/ACRE3 bind to TGGnCGGTG and TGGTCGGT/C, which are consistent with our results. Furthermore, ORA47/ACRE3 was shown to bind to the sequence TGCGACCAAA in the promoter of *MYC2*, AACGCCCAAT in the promoter of *PIN2*, and TACGACCTAA in the promoter of *ERF1* (Chen et al. 2016), although they do not fit the GnCGGT sequence.

In contrast to the ability of ACRE to bind both CMSRE-1 and DRE, DREB bound only to DRE. However, DREB proteins also recognize sequences other than DRE. For example, *Arabidopsis* DREB such as DREB1A, DREB2A, TINY, and WIND1/RAP2.4 were shown to bind to ERE (GGCGGCT in the opposite strand) (Sakuma et al. 2002; Lin et al. 2008; Sun et al. 2008).

3D structure of the AP2 domain in the complex with DNA has been well characterized with some ERF-type AP2 TFs by NMR and X-ray crystallography. For example, Shoji et al. (2013) revealed the relationship between amino acid residues of the AP2 DNA-binding domain and divergent DNA-binding specificities. Moreover, Chen et al. (2020) visualized the entire structure of the ERF96-DNA complex in detail. However, little is known about the structure of the DREB AP2 domain. It has been reported that the 14th valine (Val14) and the 19th glutamic acid (Glu19) in the AP2 domain are important for the DNA-binding activity of DREB1A and DREB2A, and the 37th alanine is important for the DNA-binding activity of both DREB and ERF subfamilies (Cao et al. 2001; Sakuma et al. 2002; Liu et al. 2006). However, the Glu19 was replaced by a Leu residue in *Arabidopsis* ACREs and morning glory InACRE1 and InACRE2, all of which can bind to CMSRE-1. Hence, we suspected that this amino acid difference would result in specificity of the binding for the CMSRE-1 sequence. Therefore, we examined the ability of transcriptional activation by modified forms of ACRE and DREB in which the 19th amino acids were exchanged with each other in a protoplast transient expression system. ACRE2 (L29E) acted on both CMSRE and DRE like ACRE2, but in the case of CMSRE-1, the level of activation by ACRE2 (L29E) was significantly reduced. This might have been due to a decrease in the affinity of ACRE (L29E) for CMSRE-1. Conversely, DREB1A (E68L) acted on DRE with the same activation level as DREB1A, and did not act on CMSRE-1. It is thought that the amino acid difference at the 19th position in the AP2 domain strongly affects the specificity of the binding sequence, but does not alone determine the sequence specificity. Other conserved amino acid residues, such as Ser at the 22nd and Phe at the 41st, in the AP2 domain might be involved in binding sequence specificity, and it is expected that these amino acid residues will be identified in order to elucidate the difference in functions among members of the DREB subfamily. In transient co-expression in *Arabidopsis* protoplasts, ACRE1 and ACRE2 activated the transcription from sugar-responsible minimal promoters, and mutation in CMSRE-1 resulted in a significant reduction in the induction level, but transactivation of LUC activity still remained (Fig. 1). This is thought to have been because ACRE1 and ACRE2 weakly bound to the mutated CMSRE-1 or to the sequence other than the CMSRE-1 within the promoter region, and the existence of interactions with other endogenous TFs such as B3 and ATHB family isolated by the same screening of ACRE1 and ACRE2 (Suppl. Table S1, Morikami et al 2005b).

There are 15 members in the DREB A-5 (ERF group II) subfamily in *Arabidopsis*. Among them, functional characterization has reported only for six members of DEARs which have the EAR-motif known to suppress transcription and regulate defense and freezing stress responses (Tsutsui et al. 2009), and ORA47/ACRE3 whose expression is strongly induced by methyl jasmonate and wounding, along with regulating jasmonic acid and abscisic acid biosynthesis and signaling (Chen et al. 2016). There is no information about TFs belonging to the ACRE clade other than ORA47/ACRE3.

The mRNA level of ACREs is not changed by sugar supply in *Arabidopsis* leaves (Suppl. Fig. S8). Therefore, the function of ACREs in the regulation of sugar-responsive gene expression may be regulated by post-translational modifications, such as phosphorylation, or by interactions with other factors. We confirmed that the overexpression of ACREs activated the expression of endogenous sugar-responsible *Atß-Amy* gene in *Arabidopsis* and enhanced its expression after sugar treatment. The overexpression also upregulated the sugar-responsible *sporamin* promoter:*LUC* in transgenic *Arabidopsis* leaves. These results are consistent with the possibility of post-translational modification in ACRE proteins.

Since *sporamin* and *ß-amylase* are genes of sweet potato, it is expected that the orthologs of ACRE are present in sweet potato. However, the sweet potato genome sequence has not been determined, so we decided to use Japanese morning glory, which is in the same genus as sweet potato. We successfully identified four orthologs of ACRE and named them InACRE1 to 4. InACRE1, InACRE2, and InACRE3 bound not only to the DRE sequence but also to the CMSRE-1 sequence, and it was found that InACRE1 and InACRE2 have transcriptional activation ability like ACRE1, 2, and 3. The closeness in the phylogenetic tree suggests that ACRE orthologs encoded by *INIL14g06668* and *INIL01g36561*, which could not be analyzed in this study, could also act on CMSRE-1. InACRE4 has quite low DNA-binding activity as determined by EMSA, exhibited sufficient transcriptional repression activity via all sequences tested in the transient expression system. This is probably because the recombinant MBP-InACRE4 fusion protein used for EMSA had relatively weak DNA-binding activity. However, these results are consistent with the presence of the EAR motif at the C-terminus of InACRE4, similar to findings in DEARs of *Arabidopsis*. Anyway, the mechanism regulating gene expression by the combination of ACRE and CMSRE-1 appears to be conserved between *Arabidopsis* and Ipomoea, and the expression of the sugar-responsible minimal promoter of *ß-amylase* and *sporamin* with the CMSRE-1 sequence is functionally induced by sugar in several plant species, such as tobacco and *Arabidopsis*. Moreover, the expression of the sugar-responsible minimal promoter of *sporamin* is higher in storage roots (Honma and Yamakawa 2019). These findings suggest that this mechanism is involved in the regulation of the sugar-responsive gene expression and is widely conserved across plant species. ACRE3/ORA47 has been shown to function in the jasmonic acid and ABA signaling pathways (Chen et al. 2016), and several genes such as *VEGETATIVE STORAGE PROTEIN* of soybean and *Arabidopsis* are known to respond to both sugar and jasmonic acid (Mason and Mullet 1990; Berger et al 1995). As such, it is expected that the mechanism regulating gene expression through the ACRE and CMSRE-1 modules functions downstream of converging plant hormone and/or sugar signaling pathways among multiple existing and complex sugar signal pathways. Therefore, this mechanism might be involved in crosstalk between sugar- and jasmonate-signaling pathways. This might be due to the redundancy of ACRE and ACRE homologs, so it anticipated that the analysis of triple mutant for ACRE1, ACRE2, and ORA47/ACRE3, which actually act on CMSRE-1, would reveal the physiological roles of ACREs in plants.

### Supplementary Information

Below is the link to the electronic supplementary material.

Supplementary file1 (PDF 1043 KB)

## Data Availability

The datasets generated and/or analyzed during the current study are available from the corresponding author on reasonable request.
