# Supplementary material for: ACRE, a class of AP2/ERF transcription factors, activates the expression of sweet potato ß-amylase and sporamin genes through the sugar-responsible element CMSRE-1
Source: Plant Mol Biol. 2024 May 7;114(3):54. doi: 10.1007/s11103-024-01450-z (PMC11076338; doi:10.1007/s11103-024-01450-z)
Supplement: Supplementary file 1 — Supplementary file1 (PDF 1043 KB) [file 11103_2024_1450_MOESM1_ESM.pdf]

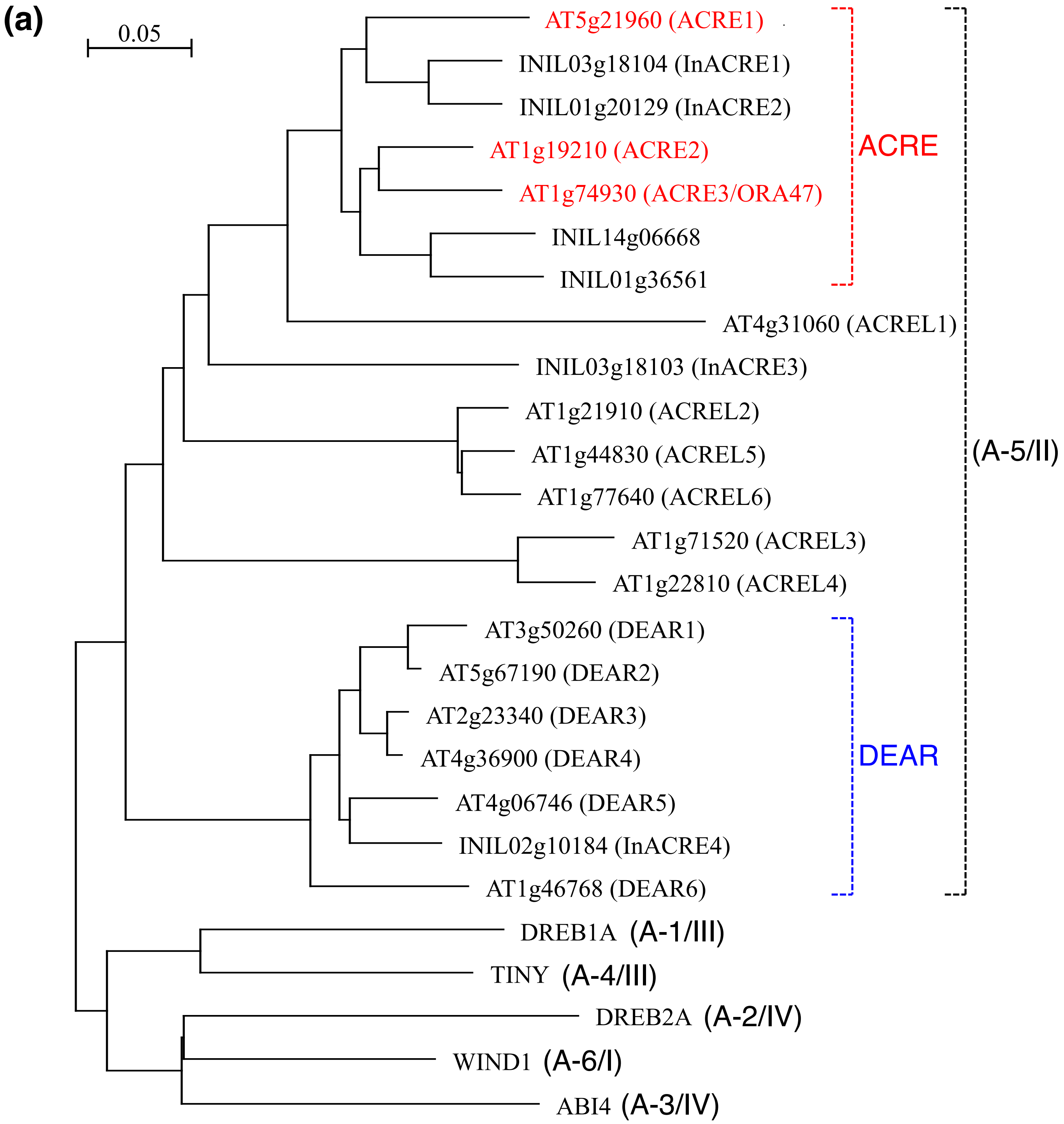

(a)
0.05
AT5g21960 (ACRE1)
INIL03g18104 (InACRE1)
INIL01g20129 (InACRE2)
AT1g19210 (ACRE2)
AT1g74930 (ACRE3/ORA47)
INIL14g06668
INIL01g36561
ACRE
AT4g31060 (ACREL1)
INIL03g18103 (InACRE3)
AT1g21910 (ACREL2)
AT1g44830 (ACREL5)
AT1g77640 (ACREL6)
AT1g71520 (ACREL3)
AT1g22810 (ACREL4)
(A-5/II)
AT3g50260 (DEAR1)
AT5g67190 (DEAR2)
AT2g23340 (DEAR3)
AT4g36900 (DEAR4)
AT4g06746 (DEAR5)
INIL02g10184 (InACRE4)
AT1g46768 (DEAR6)
DEAR
DREB1A (A-1/III)
TINY (A-4/III)
DREB2A (A-2/IV)
WIND1 (A-6/I)
ABI4 (A-3/IV)

(b)

```
                                           *       *                   *
AT5G21960(ACRE1)      KYTGVRKRKWGKWVAEIRLPNSRDRIWLGSFDSAEKAARAFDAALYCLRGPGAR----FNFP
AT1G19210(ACRE2)      KYKGVRKRKWGKWVSEIRLPNSRERIWLGSYDTPEKAARAFDAALYCLRGNNAK----FNFP
AT1G74930(ACRE3)      KYKGVRKRKWGKWVSEIRLPHSRERIWLGSYDTPEKAARAFDAAQFCLRGGDAN----FNFP
AT4G31060(ACREL1)     CYRGVRKRSWGKWVSEIRVPKTGRRIWLGSYDAPEKAARAYDAALFCIRGEKGV----YNFP
AT1G21910(ACREL2)     KYKGVRMRSWGSWVSEIRAPNQKTRIWLGSYSTAEAAARAYDVALLCLKGPQAN----LNFP
AT1G71520(ACREL3)     KYKGIRRRKWGKWVSEIRVPGTRQRLWLGSFSTAEGAAVAHDVAFYCLHRPSSLDDESFNFP
AT1G22810(ACREL4)     KYKGIRRRKWGKWVSEIRVPGTRDRLWLGSFSTAEGAAVAHDVAFFCLHQPDSL--ESLNFP
AT1G44830(ACREL5)     KYKGVRMRSWGSWVSEIRAPNQKTRIWLGSYSTAEAAARAYDAALLCLKGSSANN---LNFP
AT1G77640(ACREL6)     KYKGVRMRSWGSWVTEIRAPNQKTRIWLGSYSTAEAAARAYDAALLCLKGPKAN----LNFP

At3g50260(DEAR1)      PFKGIRMRKWGKWVAEIREPNKRSRLWLGSYSTPEAAARAYDTAVFYLRGPTAT----LNFP
At5g67190(DEAR2)      PYKGIRMRKWGKWVAEIREPNKRSRLWLGSYSTPEAAARAYDTAVFYLRGPTAR----LNFP
At2g23340(DEAR3)      PYKGIRMRKWGKWVAEIREPNKRSRIWLGSYATPEAAARAYDTAVFYLRGPSAR----LNFP
At4g36900(DEAR4)      PYKGIRMRKWGKWVAEIREPNKRSRIWLGSYSTPEAAARAYDTAVFYLRGPSAR----LNFP
At4g06746(DEAR5)      RYKGIRMRKWGKWVAEIREPNKRSRIWLGSYKTAVAAARAYDTAVFYLRGPSAR----LNFP
At1g46768(DEAR6)      PYRGIRRRKWGKWVAEIREPNKRSRLWLGSYTTDIAAARAYDVAVFYLRGPSAR----LNFP

INIL03g18104(InACRE1) KYRGVRKRKWGKWVSEVRLPNSRERIWLGSFDSPEKAARAFDAALFCLRGETAN----FNFP
INIL01g20129(InACRE2) KYRGVRKRNWGKWVSEIRLPNSRERIWLGSFDSPEKAARAFDAALFCLRGKNAN----FNFP
INIL14g06668          KYTGVRKRKWGKWVSEIRLPNSRERIWLGSYNAPEKAARAFDVAQYCLRGPTAR----FNFP
INIL01g36561          KYKGVRKRKWGKWVSEIRLPNSRERIWLGSYNTAEKAARAFDVAQFCLRGPTAQ----LNFP
INIL03g18103(InACRE3) KFKGVRMRKWGKWVAEVRQPNSRDRIWLGSYDTAEEAARAYDAAALSLRGQTAK----LNFP
INIL02g10184(InACRE4) PYKGIRMRKWGKWVAEIREPNKRSRIWLGSYSTPVAAARAYDIAVYYLRGPSAR----LNFP

DREB1A                IYRGVRRRNSGKWVCEVREPNKKTRIWLGTFQTAEMAARAHDVAALALRGRSAC----LNFA
DREB2A                SFRGVRQRIWGKWVAEIREPNRGSRLWLGTFPTAQEAASAYDEAAKAMYGPLAR----LNFP
TINY                  VYRGVRKRNWGKWVSEIREPRKKSRIWLGTFPSPEMAARAHDVAALSIKGASAI----LNFP
ABI4                  RYRGVRQRSWGKWVAEIREPRKRTRKWLGTFATAEDAARAYDRAAVYLYGSRAQ----LNLT
WIND1                 LYRGVRQRHWGKWVAEIRLPRNRTRLWLGTFDTAEEAALAYDKAAYKLRGDFAR----LNFP
                       : *:* *  *.** *:* *    * ***:: :   ** *.* *   :    .     *:.
```

Supplemental Figure S1 Phylogenetic tree of the DREB subfamily of *Arabidopsis thaliana* and the ACRE homologs of morning glory.

The amino acid sequences of AP2 domain were aligned by ClustalW on the DDBJ website (https://clustalw.ddbj.nig.ac.jp/). (a) The phylogenetic tree was constructed using the NJ method. Classifications of groups by Sakuma et al. (2002) and Nakano et al. (2006) are indicated in parentheses with a dash line. (b) Alignment of amino acid residues corresponding to AP2 domain. Dashes indicate gaps in the amino acid sequences introduced to optimize alignment. The dark gray and light gray background represents fully and highly conserved amino acid residues, respectively. The yellow background represents conserved leucine residues in ACREs and InACREs that can bind to CMSRE-1. The asterisks represents the 14th valine (Val14) and the 19th glutamic acid (Glu19) , and the 37th alanine in the AP2 domain, shown to be important for the DNA-binding activity of DREB1A and DREB2A, and both DREB and ERF subfamilies (Cao et al. 2001; Sakuma et al. 2002; Liu et al. 2006).

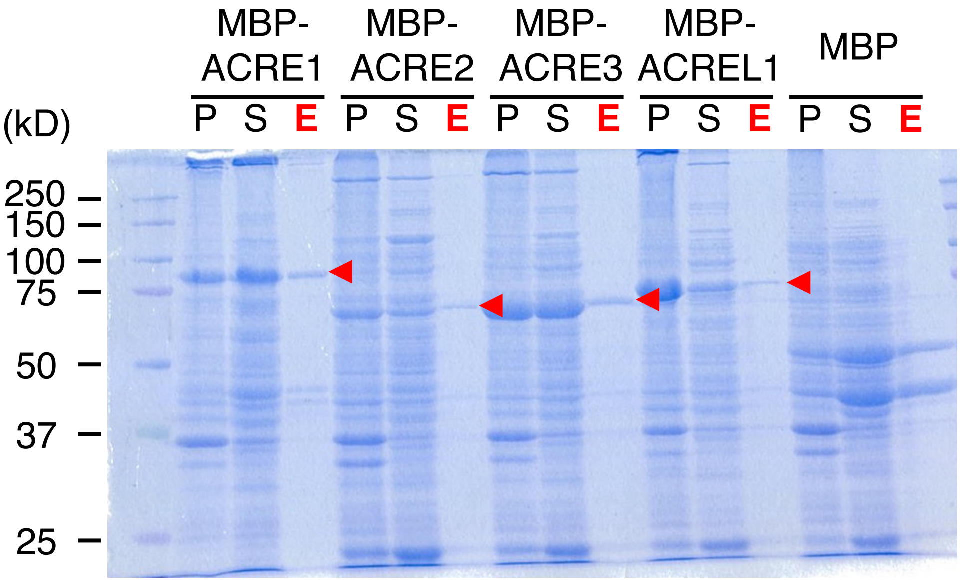

Supplemental Figure S2 Purification of the recombinant MBP-ACRE fusion proteins. Purification of the recombinant MBP-ACRE fusion protein was performed in accordance with the instruction manual for pMAL protein fusion and purification system (New England BioLabs). Equal amounts corresponding to the crude extract were applied for the resuspended pellets of *E. coli* cells (P), supernatant after extraction (S), and elute (E), used for EMSA. The red arrowhead indicates the band corresponding to the MBP-fusion protein.

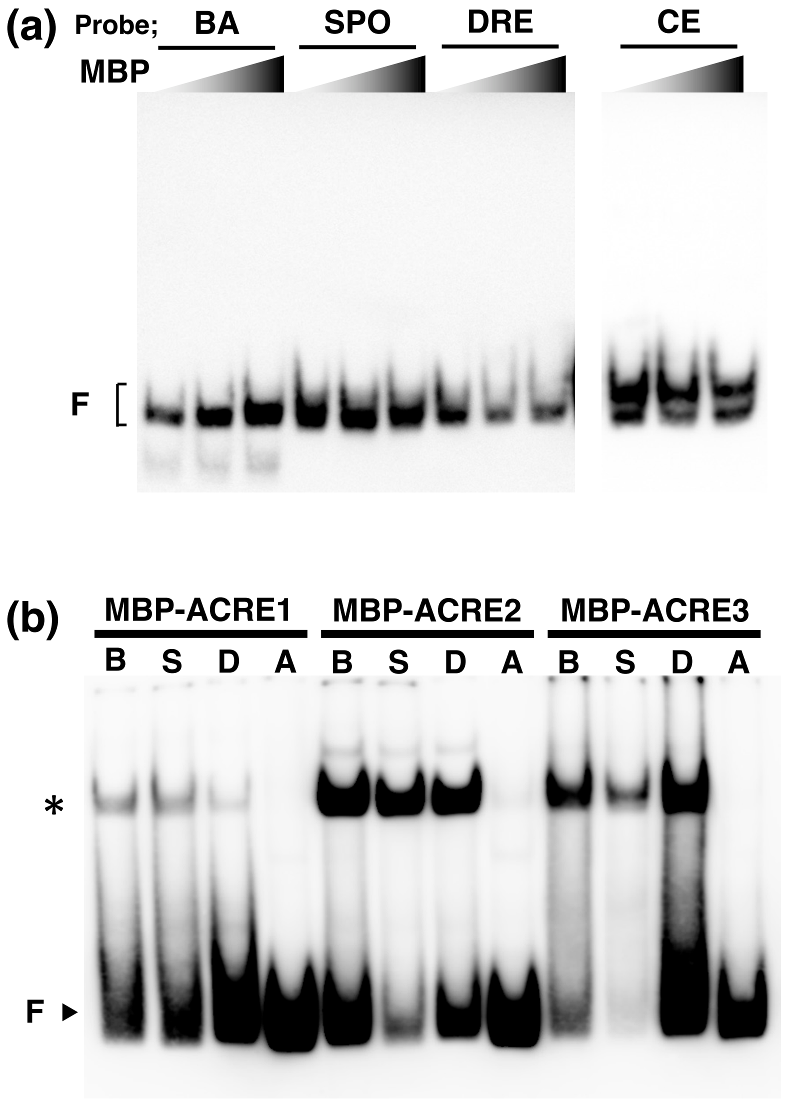

(c) A(AtBA,Atβ-Amy);
ctagTTCAAATGGACcGCTGAAt

Supplemental Figure S3 MBP-ACRE1, 2, and 3 bind to the CMSRE-1 of ß-amylase and sporamin A1, and the DRE, but not the CMSRE1-like element of ß-amylase of Arabidopsis.
(a) MBP protein alone does not have binding activity. (b) The DNA-binding activity of MBP-fusion proteins with ACRE was analyzed by EMSA using BA (B), SPO (S), DRE (D), and AtBA oligonucleotides (A) as probes. Asterisk and arrowhead indicated by F show the position of the shifted band and free probe, respectively. (c) The sequences of AtBA oligonucleotides used for EMSA.

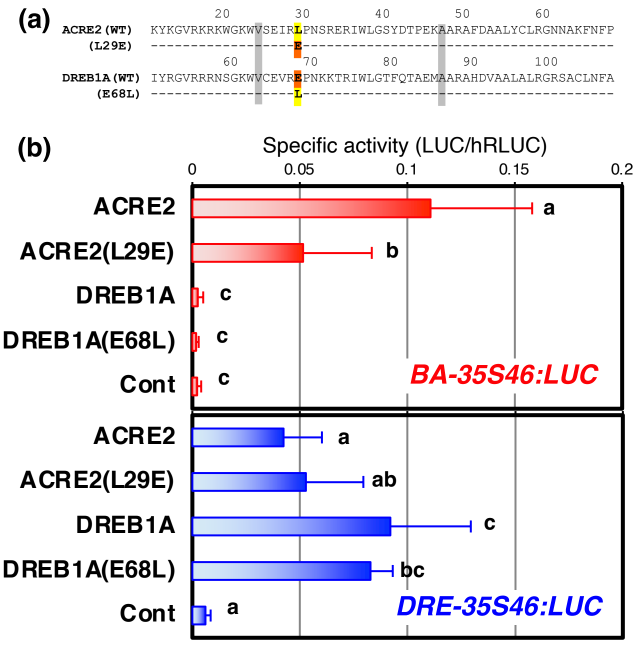

Supplemental Figure S4 The effect of amino acid substitution at AP2 domain to transactivation.
(a) Amino acid residues of AP2 domain for ACRE2, DREB1A and their amino acid substituted forms.
(b) Transactivation of BA-35S46:LUC (top) and DRE-35S46:LUC (bottom) by the co-expression of wild type or amino acid substituted ACRE2 and DREB1A.
The LUC activity in each assay was normalized to the hRLUC activity, and normalized activity (LUC/hRLUC) is expressed relative to values obtained with the empty vector. Results represent the means of six independent experiments, with the error bar representing SD. Values that were significantly different from one another according to Tukey's multiple comparison test are indicated by different letters ($P < 0.05$).

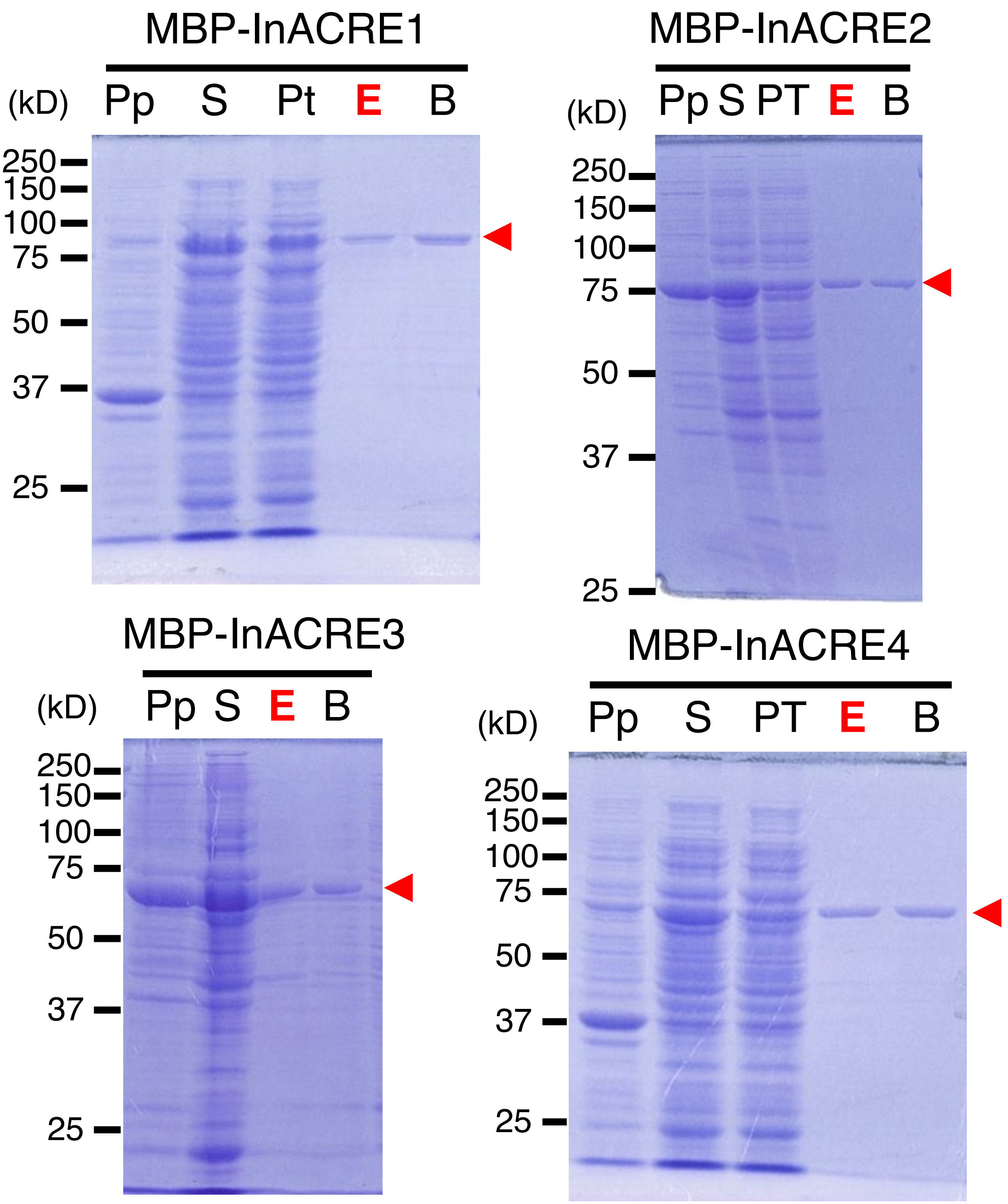

Supplemental Figure S5 Purification of the recombinant MBP-InACRE fusion proteins. Purification of the recombinant MBP-InACRE fusion protein was performed as shown in figure S2. Equal amounts corresponding to the crude extract were applied for resuspended pellets of *E. coli* cells (P), supernatant after extraction (S), and path-through after binding with amylose resin (PT), elute (E), used for EMSA, and resuspended amylose resin (B). The red arrowhead indicates the band corresponding to the MBP-fusion protein.

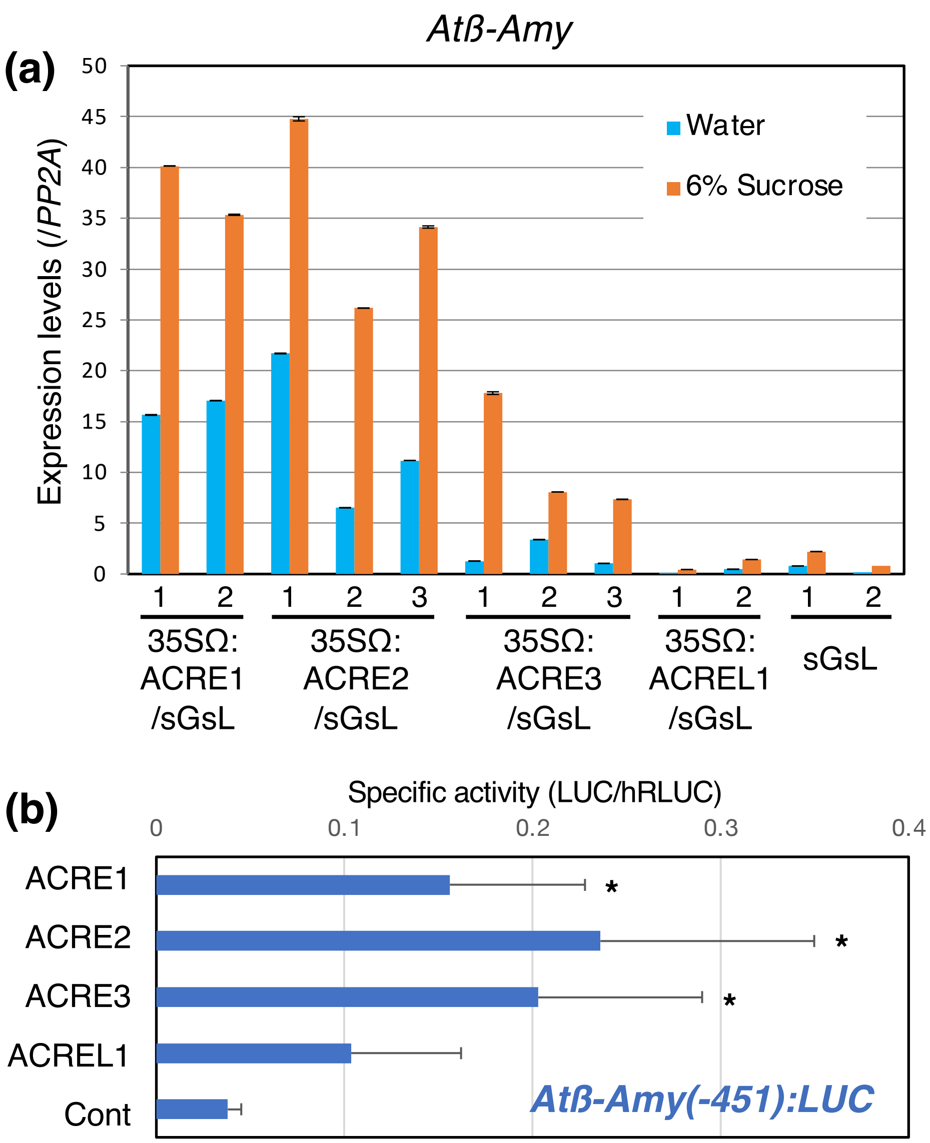

Supplemental Figure S6 Effect of ACRE over-expression on the sugar-responsible gene expression

(a) Expression of *Atβ-amylase* gene in the ACRE- and ACREL1-overexpression lines. Leaf-petioles of 3-week-old seedlings were isolated and incubated in water (blue bars) or 5% sucrose (orange bars) for 2 days and then subjected to RT-qPCR analysis of *Atβ-Amy* transcripts. Measured values of two to three independent plants in each class are indicated separately as levels relative to PP2A (At1g13320). For each plant, two samples consisting of three leaves were averaged and shown with standard deviation.
(b) Transactivation of *Atβ-Amy(-451):LUC* by co-expression of ACRE2 in Arabidopsis protoplasts. The average of relative activity (LUC/hRLUC) in 4 independent experiments is shown with the error bar representing standard deviation. Asterisks indicate significant change at $P < 0.05$ (Student's T test) against the control.

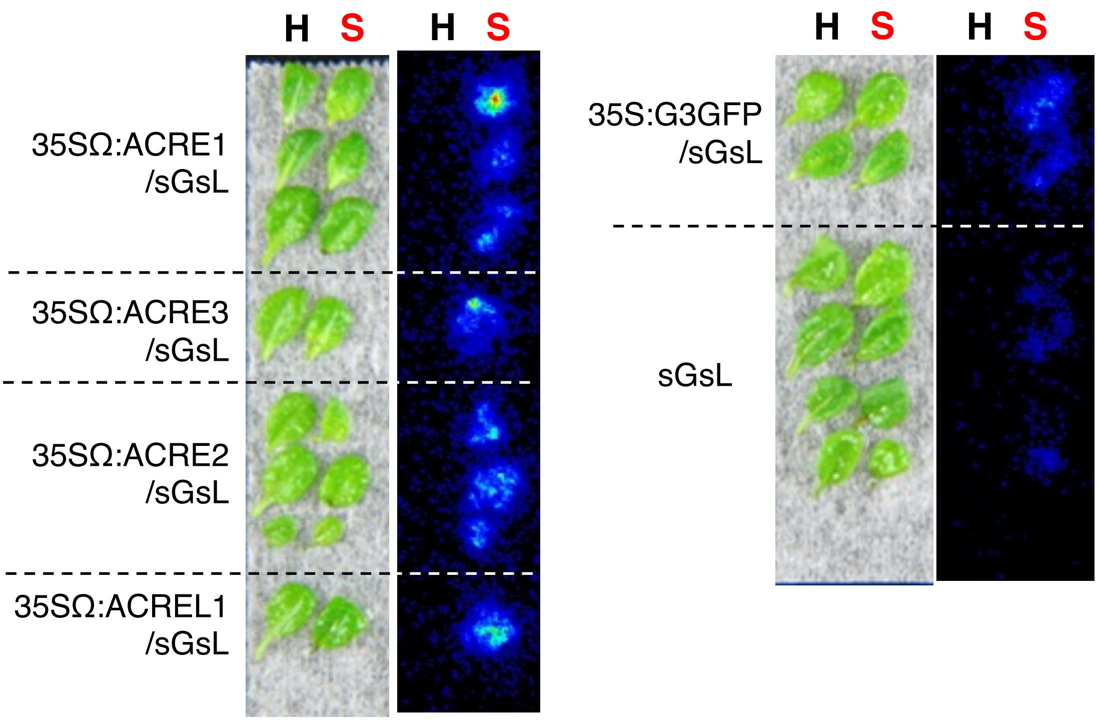

Supplemental Figure S7 Luminescence image of LUC activity in the ACRE- and ACREL1-overexpressing sGsL lines.
Leaf-petioles of 3-week-old seedlings were incubated in water (H) or 6% sucrose (S) for 24 h, then they were sprayed with luciferin solution prior to detection of luminescence with a high resolution photon counting camera.

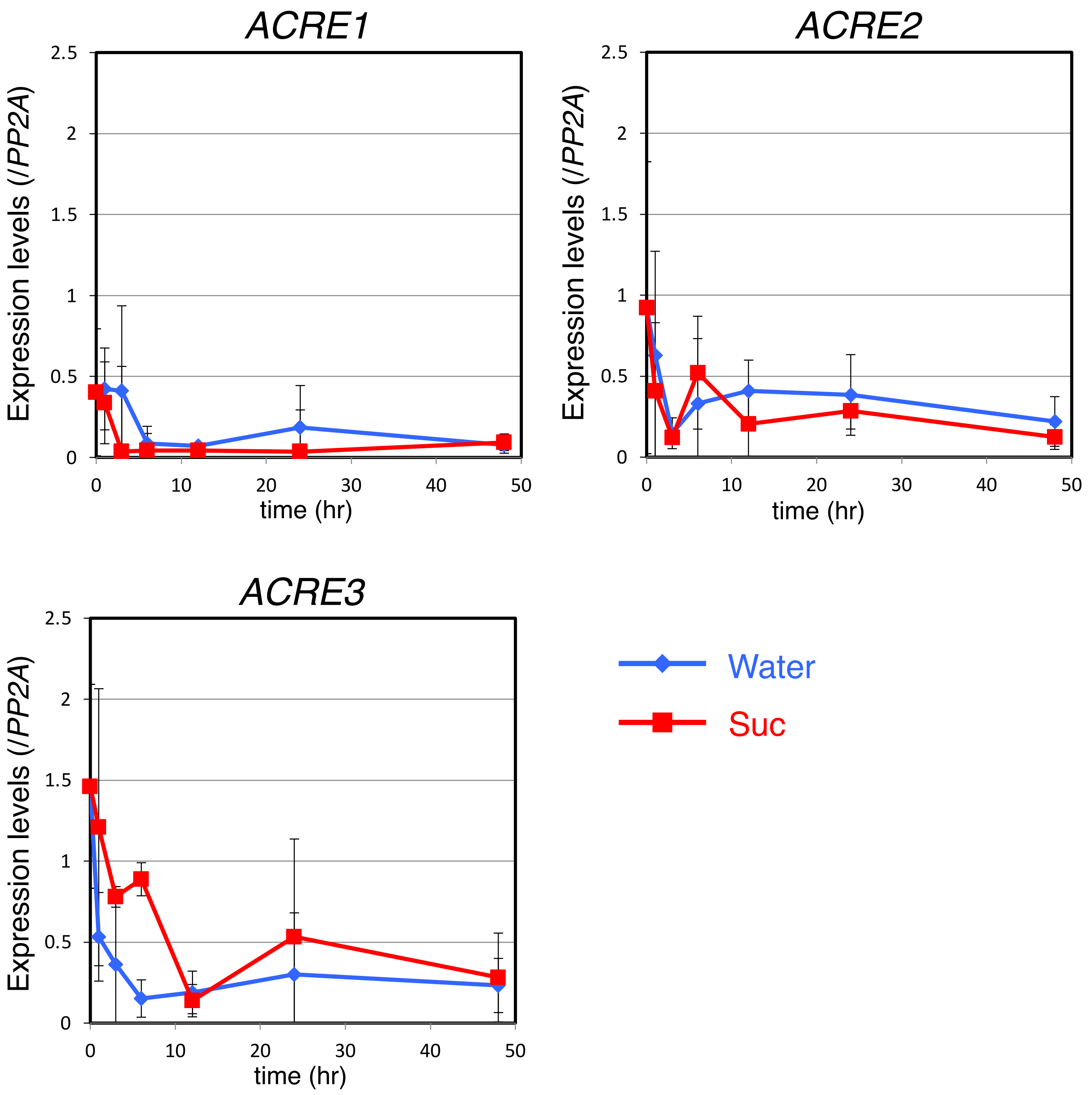

Supplemental Figure S8 Expression of ACRE genes in response to sugar supply.
Leaf-petioles of 3-week-old Arabidopsis seedlings incubated in water for 24 h and then in water or 5% sucrose for a further time indicated and then subjected to RT-qPCR analysis of transcripts. The means of the amounts relative to PP2A (At1g13320) in three independent samples from three leaves, are shown with standard deviation.

Supplemental Table S1 Positive clones of yeast-one hybrid screening.
The cDNA sequences of clones from yeast strains harboring the 2x430HISi (a) and 4x495:HISi (b), respectively, that grew with good reproducibility on the selective medium were determined

(a)

MIPS	gene	clone
AT5G65310	ATHB5, ATHB-5, HB5	8
AT3G26790	FUS3	5
AT1G28300	LEC2	4
AT2G22430	ATHB6, HB6	4
AT5G21960	Integrase-type DNA-binding superfamily protein	4
AT4G36740	HB-5, ATHB40, HB40	2
AT4G00730	ANL2, AHDP	2
AT1G19210	Integrase-type DNA-binding superfamily protein	1
AT1G31320	LBD4	1
AT3G06740	GATA15	1
AT4G00270	DNA-binding storekeeper protein-related transcriptional regulator	1
AT5G15150	ATHB-3, HAT7, ATHB3, HB-3	1
total		34

(b)

MIPS	gene	clone
AT1G09030	NF-YB4	1
AT1G19210	Integrase-type DNA-binding superfamily protein	1
AT1G50680	AP2/B3 transcription factor family protein	1
AT1G69690	TCP family transcription factor	1
AT2G21230	Basic-leucine zipper (bZIP) transcription factor family protein	1
AT2G21650	MEE3, ATRL2, RSM1	1
AT2G23340	DEAR3	1
AT2G37590	ATDOF2.4, DOF2.4	1
AT2G46680	ATHB-7, ATHB7, HB-7	1
AT3G25890	Integrase-type DNA-binding superfamily protein	1
AT3G62420	ATBZIP53, BZIP53	1
AT3G66656	AGL91	1
AT4G00940	Dof-type zinc finger DNA-binding family protein	1
AT4G16780	ATHB-2, HAT4, ATHB2, HB-2	1
AT4G36730	GBF1	1
AT5G07700	MYB76, AtMYB76	1
AT5G17430	BBM	1
AT5G40330	MYB23, ATMYB23, ATMYBRTF	1
AT5G43650	BHLH92	1
AT5G53900	Serine/threonine-protein kinase WNK (With No Lysine)-related	1
AT5G61930	APO3	1
AT5G67190	DEAR2	1
total		22

Supplemental Table S2 The primer sequences used for the cloning and the construction of vectors.

		Sequence (5' to 3')
ACRE3	AT1G74930attB1	GGGGACAAGTTTGTACAAAAAAGCAGGCTCAATGGTGAAGCAAGCGATGA
	AT1G74930attB2	GGGGACCACTTTGTACAAGAAAGCTGGGTAAAAATCCCAAAGAATCAAAGATTCATCTA
ACREL1	AT4G31060attB1	GGGGACAAGTTTGTACAAAAAAGCAGGCTTAATGCCACCCTCTCCTCCTA
	AT4G31060attB2	GGGGACCACTTTGTACAAGAAAGCTGGGTAGATTAGATTGTTTATCCAATCAATGTCC
ACREL2	AT1G21910attB1	GGGGACAAGTTTGTACAAAAAAGCAGGCTCCATGGTGAAACAAGAACGCAAG
	AT1G21910attB2	GGGGACCACTTTGTACAAGAAAGCTGGGTAATTGAAACTCCAAAGCGGAATGTC
ACREL3	AT1G71520attB1	GGGGACAAGTTTGTACAAAAAAGCAGGCTACATGGATTCAAGAGACACCG
	AT1G71520attB2	GGGGACCACTTTGTACAAGAAAGCTGGGTAAACGAGATCGTCTTCAAGATAATC
ACREL5	AT1G44830attB1	GGGGACAAGTTTGTACAAAAAAGCAGGCTCTATGGTGAAAACACTTCAAAAGACACC
	AT1G44830attB2	GGGGACCACTTTGTACAAGAAAGCTGGGTAGCAGAAGTTCCATAATCTGATATCTCC
InACRE1	InACRE1attB1	GGGGACAAGTTTGTACAAAAAAGCAGGCTTAATGGTGAAACCCAAAAGACAAACTG
	InACRE1attB2	GGGGACCACTTTGTACAAGAAAGCTGGGTAAAAATTCCAAAGAAAAGAATCTTGTGAAG
InACRE2	InACRE2attB1	GGGGACAAGTTTGTACAAAAAAGCAGGCTTAATGGTGAAACCCGAGAAAG
	InACRE2attB2	GGGGACCACTTTGTACAAGAAAGCTGGGTAGAAATTCCAGAGGAATGATCCGTC
InACRE3	InACRE3attB1	GGGGACAAGTTTGTACAAAAAAGCAGGCTCCATGCCAGTCAAACTCCAC
	InACRE3attB2	GGGGACCACTTTGTACAAGAAAGCTGGGTAGAGATAGTGCTGCATTTGGGATG
InACRE4	InACRE4attB1	GGGGACAAGTTTGTACAAAAAAGCAGGCTCTATGGAGATGGAGGGTCAG
	InACRE4attB2	GGGGACCACTTTGTACAAGAAAGCTGGGTACCAGTAATCAACATCCGGGTC

Supplemental Table S3 The primer sequences used for the expression analysis by RT-qPCR.

		Sequence (5' to 3')
ACRE1	ACRE1+351F	GGCACGTGGGGAAATTAATAGTGG
	ACRE1+446R	CCTCCCATATTAAAGGCCAATACG
ACRE2	ACRE2+312F	AGGATACGAGATACGGCAAGAATC
	ACRE2+411R	AACCCAGGGAATAACCCAAACTCC
ACRE3	ACRE3+228F	CCTAATAATCCACCGTCGATCTCC
	ACRE3+339R	ATCCGGGTACTAAACCCGATTC
PP2A	PDF2 cds+934F	AACCCTGAACTCGCTATCCAGCACA
	PDF2 cds+1065R	TGTTGCATCCTTACCCAAGACTGGA
Atß-Amy	Atß-Amy-F	CATGAGATTGTGCCGTTGAA
	Atß-Amy-R	TCGGTTTCAGAGTCCCACTT
